# Ultra-Rapid Warming Yields High Survival of Mouse Oocytes Cooled to −196°C in Dilutions of a Standard Vitrification Solution

**DOI:** 10.1371/journal.pone.0036058

**Published:** 2012-04-27

**Authors:** Shinsuke Seki, Peter Mazur

**Affiliations:** 1 Fundamental and Applied Cryobiology Group, Department of Biochemistry and Cellular and Molecular Biology, The University of Tennessee, Knoxville, Tennessee, United States of America; 2 Tokyo University of Marine Science and Technology, Tokyo, Japan; Ottawa Hospital Research Institute and University of Ottawa, Canada

## Abstract

Intracellular ice is generally lethal. One way to avoid it is to vitrify cells; that is, to convert cell water to a glass rather than to ice. The belief has been that this requires both the cooling rate and the concentration of glass-inducing solutes be very high. But high solute concentrations can themselves be damaging. However, the findings we report here on the vitrification of mouse oocytes are not in accord with the first belief that cooling needs to be extremely rapid. The important requirement is that the warming rate be extremely high. We subjected mouse oocytes in the vitrification solution EAFS 10/10 to vitrification procedures using a broad range of cooling and warming rates. Morphological survivals exceeded 80% when they were warmed at the highest rate (117,000°C/min) even when the prior cooling rate was as low as 880°C/min. Functional survival was >81% and 54% with the highest warming rate after cooling at 69,000 and 880°C/min, respectively. Our findings are also contrary to the second belief. We show that a high percentage of mouse oocytes survive vitrification in media that contain only half the usual concentration of solutes, *provided* they are warmed extremely rapidly; that is, >100,000°C/min. Again, the cooling rate is of less consequence.

## Introduction

The ability to cryobiologically preserve mammalian sperm and preimplantation embryos has played a central role in assisted reproduction in women, in improving the genetic quality of livestock, and in the maintenance of mutant and transgenic lines of mice and other mammals [1]. The successful cryopreservation of mammalian embryos was first reported in 1972 for mice [2]. More recently, the cryopreservation of the human oocyte has become a matter of intense interest [3]. First, it would permit women to delay the onset of child bearing without adverse consequences. Second, it would permit women who face the daunting prospects of becoming sterile from chemotherapy and radiation therapy to subsequently give birth to children. Third, the preservation of unfertilized oocytes does not create the ethical and legal problems that can occur with frozen embryos. These are the chief reasons for the interest; but unfortunately, the results so far have not matched the interest. As of the end of 2008, only about 900 babies world-wide have been derived from cryopreserved oocytes as opposed to tens of thousands that have developed from frozen embryos [4], and the procedure is still considered experimental.

A major cause of lethal injury during cryopreservation is the formation of more than a trace amount of ice within a cell. (Karlsson et al. [5] have calculated that the limiting amount of internal ice compatible with viability in hepatocytes is 2 to 4% of their water.) One route to avoid it is vitrification. In the vitrification approach, ice formation is avoided by suspending the cells in very high concentrations of solutes, including ones that permeate the cell, and cooling them at high rates to temperatures below −100°C. As a result, the water in the system is converted from a liquid to a glass with no ice formation. The approach also requires high warming rates to ensure that the system does not convert from glass to ice during warming.

There have been two firmly held premises in the vitrification approach. One is that avoiding ice formation in cells and obtaining high survivals demands the highest of cooling rates. Consequently, a series of devices have been developed over the past decade that achieve cooling rates of ≥10,000°C/min by permitting the manipulation of very small volumes of oocyte suspensions. These include electron microscope grids [6], nylon mesh [7], open-pulled straw (OPS) [8], cryoloop [9], microdrop method [10], [11], and Cryotops [12], [13].

Some authors have noted almost parenthetically that rapid warming is also necessary to prevent the vitreous water in cells from crystallizing during warming. But apart from a few scattered reports, only recently has it been experimentally demonstrated that the warming rate is much the more critical determinant of whether mouse oocytes survive vitrification procedures, and not the cooling rate [14].

The second premise in the vitrification approach is that the vitrification solution in which the cells are suspended must have a very high concentration of a mixture nonelectrolytic solutes. We have used EAFS 10/10, a solution developed by Pedro et al. [15], where E, A, F, and S refer to ethylene glycol (EG), acetamide, Ficoll, and sucrose. The mass composition (Table 1, top row) is 3.23 molal EG, 3.27 m acetamide, 0.72 m sucrose, 0.15 m salt, and 20.7 wt. % Ficoll (24% w/v). The total molality is 7.37 molal, of which 6.5 molal is permeating (EG and acetamide), and the remainder non-permeating. This composition is relatively typical of most vitrification solutions in containing mixtures of permeating and nonpermeating solutes. These very high concentrations can have serious consequences. First, they can be chemically toxic to the cells. Second, they produce major cell osmotic dehydration that may itself be damaging. In EAFS 10/10, for example, in approximately 2 min, the oocyte water contents will drop to near 0.3/7.4 or ∼5% of their isotonic value. However, as they approach equilibrium over the next ∼20 min, their water volume will gradually increase to 28% as the permeating solutes enter (Table 1, column M) [16]). Ordinarily, if one attempts to reduce this problem by reducing the concentration, one faces the “catch −22" problem faced by Ulysses that the farther from Charybdis, the nearer is Scylla; that is, slight reductions in solute concentration greatly increase the cooling rate required to achieve vitrification and greatly increase the warming rate needed to prevent devitrification [17].

**Table 1:** Derivative solute concentrations in various dilutions of the EAFS10/10 vitrification solution.

(A)	(B)	(C)	(D)	(E)	(F)	(G)	(H)	(I)	(J)	(K)	(L)	(M)
Relative	Total	Total	Molality	Molality	Molality	Molality	Molar	Molar	Molar	Molar	Osmol	Rel. vol
concn,	mass	volume	EG	acet.	sucros.	NaCl	EG	acet.	sucros.	NaCl	non-perm.	cell
EAFS	(g)	(ml)										water
1×	11.52	10	3.23	3.27	0.72	0.15	1.345	1.362	0.292	0.062	0.996	0.277
0.875×	11.33	10	2.57	2.6	0.57	0.15	1.217	1.233	0.272	0.056	0.849	0.325
0.75×	11.14	10	2.01	2.04	0.45	0.15	1.079	1.093	0.241	0.051	0.726	0.38
0.5×	10.76	10	1.15	1.16	0.26	0.15	0.771	0.78	0.172	0.035	0.533	0.518
0.33×	10.51	10	0.7	0.71	0.16	0.15	0.538	0.544	0.12	0.024	0.436	0.633

Columns D–G. The molalities are calculated as Columns N-Q of Table 2/MW of the respective solute.

Columns H–K: The molarities are calculated as 100× Column B/Wt% in Column G-K of Table 2/MW of the solute.

Column L: The nonpermeating solutes are sucrose and PBS (we ignore the very small contribution of the Ficoll and BSA.) Their combined osmolality is Column F+0.276. We assume the osmolality of PBS to be equal to that of the same molality of NaCl. The osmolality of NaCl = 2φm where 2 is the number of species into which the molecule dissociates and φ is the osmotic coefficient.

Column M: The volume of water in the oocytes after equilibration with the external medium relative to the volume of water in an isotonic cell. It is 0.276/Column L.

Based on our previous demonstration that a very high warming rate and not a high cooling rate was the essential element to surviving vitrification procedures in full strength EAFS [16], we hypothesized for the present study that the use of very high warming rates might also permit the use of more dilute vitrification solutions. As we report, that has turned out to be the case.

## Materials and Methods

### Collection of Oocytes

Mature female ICR mice were induced to superovulate with intraperitoneal injections of 5 IU of equine chorionic gonadotropin (eCG) and 5 IU of human chorionic gonadotropin (hCG) (Sigma, St. Louis) given 48 h apart. Ovulated unfertilized oocytes were collected from the ampullar portion of the oviducts at 13 h after hCG injection and were freed from cumulus cells by suspending them in modified phosphate-buffered saline (PB1) containing 0.5 mg/ml hyaluronidase followed by washing with fresh PB1 medium.

### Ethics Statement

All the procedures involving mice were carried out under the University of Tennessee Institutional Animal Care and Use Committee protocol 911-0710, approved 9 July 2010 and renewed 3 May 2011. It has been assigned a pain/distress category of C.

### Composition of Vitrification Solution

The vitrification solution (EAFS 10/10) was developed by Pedro *et al.* [15] for cryopreservation of mouse oocytes at metaphase II stage. It consists of 10% (v/v) ethylene glycol (EG) and 10.7% (w/v) acetamide dissolved in a stock consisting of 30% (w/v) Ficoll 70 and 0.5 M sucrose in PB1 medium. The final concentration of sucrose and Ficoll are 0.4 M and 24% w/v (20.7 wt %), respectively. Its mass composition was given in a previous report [16] and is shown in the top row of Tables 1 and 2; namely, 3.23 molal EG and 3.27 molal acetamide as the highly permeating cryoprotectants, and 0.150 molal salts (as NaCl) and 0.720 molal sucrose as impermeable solutes, plus 20.68 weight % of Ficoll and 0.166 weight % of bovine serum albumin. We refer to this as 1× EAFS. The 20.7 wt% of Ficoll 70 corresponds to 431 g/kg water [16], which is ∼0.006 molal.

**Table 2:** Mass compositions of various dilutions of EAFS10/10 vitrification solution.

(A)	(B)	(C)	(D)	(E)	(F)	(G)	(H)	(I)	(J)	(K)	(L)	(M)	(N)	(O)	(P)	(Q)
Rel.	Volume	Mass	Mass	Total	Mass	Wt %	Wt %	Wt %	Wt %	Wt %	Wt%	Wt %	g EG/	g acet/	g sucr/	g NaCl/
Concn	1× EAFS	1× EAFS	PB1	mass	fraction	EG	Acet	salt	Ficoll	sucr	BSA	water				
EAFS	(ml)	(g)	(g)	(g)	EAFS			(g/100 g)						(kg/water)		
1×*	10	11.52	0	11.52	1	9.62	9.27	0.42	20.68	11.84	0.16	48.01	200.37	193.08	246.62	8.75
0.875×	8.75	10.08	1.25	11.33	0.89	8.56	8.25	0.37	18.4	10.53	0.14	53.75	159.25	153.45	195.99	8.75
0.75×	7.5	8.64	2.5	11.14	0.78	7.46	7.19	0.33	16.04	9.18	0.12	59.68	125.03	120.48	153.88	8.75
0.5×	5	5.76	5	10.76	0.54	5.15	4.96	0.22	11.07	6.34	0.09	72.17	71.36	68.76	87.83	8.75
0.33×	3.33	3.84	6.67	10.51	0.37	3.51	3.38	0.15	7.55	4.32	0.06	81.02	43.36	41.78	53.37	8.75

* From Table 1 of [16].

Column C: Col. B X density EAFS from [16].

Column D: Density of PB1 = 1.003; therefore volume (ml) ≒ mass (g).

Column F = Column C/Column E.

Column G = 9.62× Column F, where 9.62 is from Table 1 in [16].

Columns H-L: Wt% of solutes in 1× EAFS from Table 1 of [16] X Column F.

Column N = (G/(M/100)*10); Column O = (H/(M/100)*10); etc.

To prepare 0.875×, 0.75×, 0.50×, and 0.33× dilutions of the 1× EAFS 10/10, we added 1.25 ml, 2.5 ml, 5.0 ml, and 6.67 ml of PB1 to 8.75 ml, 7.5 ml, 5.0 ml, and 3.33 ml of the full-strength solution. The mass compositions of the resulting diluted solutions are also shown in Tables 1 and 2. The permeable solutes EG and acetamide cause a transient osmotic decrease in oocyte volume followed by a return to their original volume in less than 15 min. In contrast, the non-permeating solutes, sucrose and salts, result in the cells having a permanently lowered water content. The water contents at equilibrium are shown in the right hand column of Table 1. They are calculated from the Boyle van't Hoff law which states that the volume of water in an anisotonic medium relative to the volume of water in an isotonic medium is equal to the isotonic osmolality divided by the osmolality of nonpermeating solutes in the medium. Note that the osmolality of the NaCl is about twice the molality. This is because NaCl dissociates into Na and Cl ions each of which contribute to the osmotic pressure. Ficoll is also impermeable but because its average molecular weight is 70,000, its molality is only ∼0.006 molal. However, at high concentrations, the osmolality of such polymers ceases to be a linear function of their molality. Information in Data Sheet 18-1158-27 AA, 2001-11 published by Amersham Biosciences (Piscataway, NJ) , the manufacturer of Ficoll 70, shows that the osmolality of a 0.003 molal solution of Ficoll 70 in water is roughly four times the molality. For the 0.006 molal in EAFS 10/10, the osmolality is probably 0.06 osm, or 10-fold higher. But that is still too small to contribute significantly to the osmotic equilibrium, and we have ignored that contribution.

### Description of the Cryotop

The Cryotop (Kitazato Co., Fuji, Japan) consists of a flat rectangular leaf of polypropylene measuring 20×0.7×0.1 mm that is attached to a thin handle some 5 cm long. A photograph is shown in [18]. For a run, about 6 oocytes were transferred at 22°C to successive drops of the desired dilution of EAFS 10/10, and then 0.1 µl of the last solution along with the six oocytes was pipetted onto the rectangular leaf of the Cryotop, To minimize evaporation, the cooling of the Cryotops to −196°C was initiated within 15 to 30 sec by the methods described in the next section. In some cases the Cryotop was inserted into an outer insulating tube(s) just prior to cooling. The elapsed time between the initial exposure of the oocytes to the EAFS and the initiation of cooling was held close to two minutes.

### Achieving Various Cooling and Warming Rates with Cryotops

As indicated in Table 3, three of the seven cooling rates studied used Cryotops cooled either in LN_2_ (−196°C) (Cooling Protocol 7) or in LN_2_ vapor (∼−150°C)(Cooling Protocols 2 and 5). In two cases (Protocols 2 and 5), the Cryotop was insulated. In one case (Protocol 7) it was not. All samples on Cryotops were warmed at the highest rate of 117,500°C/min by abrupt immersion of the naked Cryotop in 2 ml of 0.5 M sucrose at 23°C. (Sucrose is an impermeant solute, the purpose of which is to provide osmotic buffering when the oocytes are diluted out of the concentrated EAFS 10/10). This means that the insulation surrounding the Cryotops in cooling protocols 2 and 5 had to be removed under or just above the LN_2_ prior to initiating warming. Cooling rates were calculated from 20°C to −120°C; warming rates from −130°C to −30°C. The upper limit of −30°C was chosen because the temperature/time curve became very curvilinear above −30°C as a result of the progressive melting of ice in the concentrated solution.

**Table 3:** Protocol of each cooling procedure.

Protocol No.	Device	The device was covered with	Cooled by	Cooling rate ±SE (°C/min)
1	Straw	1/2-ml straw + double glass*	LN_2_ vapor‡	37±0§
2	Cryotop	Cryotop-cap-straw + small glass†	LN_2_ vapor‡	95±4¶
3	Straw	None	LN_2_ vapor‡	187±6§
4	Straw	1/2-ml straw	LN_2_	522±54§
5	Cryotop	Crytop-cap-straw	LN_2_ vapor‡	876±11¶
6	Straw	None	LN_2_	1,827±214§
7	Cryotop	None	LN_2_	69,250±4,285¶

* 1/4 ml sample straw was covered with a 1/2 ml straw, a 7 mm OD ×90 mm glass tube, and a 10 mm OD ×90 mm glass tube.

† The Cryotop was covered with the Cryotop-cap-straw and a 7 mm OD ×90 mm glass tube.

‡ The straw or Cryotop were placed horizontally on a Styrofoam disk (14 cm diameter, 1.5 cm thick) floated on the surface of LN_2_ in a 41 Dewar flask for >5 min before being immersed in LN_2_.

§ The cooling rates were determined from 20 to −120°C. N = 5.

¶ The cooling rates were determined in [14].

The cooling and warming rates of samples on Cryotops were determined by cementing the junction of a 50 µm copper/constantan thermocouple to a Cryotop and overlaying it with 0.1 µl of EAFS [18]. The highest cooling and warming rates were so high that they required a special, but inexpensive computerized oscilloscope to record the temperature- time traces. An oscilloscope trace of one cooling and warming run involving an uninsulated Cryotop is shown in [18].

### Achieving Various Cooling and Warming Rate with ¼ ml Straws

Lower cooling and warming rates were achieved by placing samples in ¼ ml insemination straws (L'Aigle, Normandy, France). For cooling (Table 3), the straws were placed in LN_2_ or in LN_2_ vapor. Two of the samples in straws were cooled with insulation; two of them were cooled without insulation. All four were warmed without insulation in a water bath at 0°C or at 25°C (Table 4).

**Table 4:** Protocol of each warming procedure.

Protocol No.	Device	Air at 23–25°C		Covered by	Warmed with	Stirring*	Warming rate ±SE (°C/min)
		Time (s)	Stirring*				
1	Straw	10	-	None	Water at 0°C	+	2,170±114†
2	Straw	10	-	None	Water at 25°C	+	2,950±119†
3	Cryotop	0	-	None	Sucrose solution at 25°C	−	117,500±10,632‡

* +, yes; −, no.

† The warming rates were calculated between −70 and −35°C in [16].

‡ The warming rate was determined in [18].

The cooling and warming rates in straws were determined by placing a 36 ga thermocouple in the column of medium in the straw [14], [16].

### Post-Thawing Procedures and the Determination of Survival

The “thawed" oocytes on Cryotops were collected from the 2 ml of 0.5 M sucrose/PB1 and were then pipetted into fresh 0.5 M sucrose/PB1 solution. Approximately 10 min later, they were transferred to fresh PB1 medium lacking sucrose, and then transferred to and cultured in modified M16 medium for 2 hrs. (We simply refer to this as M16). Oocytes thawed in the straws were expelled into a watch glass containing 2 ml of 0.5 ml sucrose in PB1 [19]. From there on, the procedure was the same as with Cryotops.

Viability was initially assessed at three time points based on osmotic responsiveness and morphological normality. First, the oocytes were examined during the 10 min in sucrose/PB1. Membrane-intact oocytes were expected to shrink with time because the sucrose is hypertonic. Second and third, they were examined after being placed in M16 and after two hours incubation. They fall into two binary groups: Degenerate oocytes are clearly non-viable. The others are indistinguishable from fresh oocytes, and we know from past experiments that the plasma membranes in this latter group are intact and function normally with respect to their osmotic response to hypertonic and hypotonic media, and with respect to their ability to remain supercooled in the presence of external ice. The ability to manifest these characteristics after two hours in M16 is considered a rather stringent test of viability. Still other criteria of normality are given in [19, pp. 48–49]. They mostly deal with measures of the integrity of the plasma membrane.

### Functional Assay: In vitro Fertilization and Development to 2-Cell Embryos

In later experiments, we subjected a subset of samples of oocytes to a functional assay of viability; namely, oocytes that had yielded high survival based on the morphological/osmotic criteria. The functional assay consisted of carrying out *in vitro* fertilization (IVF) of the oocytes and determining the percentage that developed to the 2-cell stage. Sperm from male ICR mice were collected from the epididymides, transferred to M16 medium, incubated in a 5% CO_2_/95% air for 1 hr, and then transferred to fresh M16 medium. After the sperm had incubated 1 hr, the selected oocytes on Cryotops were removed from LN_2_ and, after removing any outer insulating tubes, were warmed at 117,500°C/min by abrupt immersion in 0.5 M sucrose/PB1 at 22°C. Over the ensuing 10 min, the oocytes shrank because of the hypertonic sucrose. At this point, a portion of the zona pellucida was dissected away using a surgical knife (Feather Industry Ltd., Gifu, Japan) by hand. The partially dissected (PZD) oocytes were then transferred to the suspension of sperm in M16 (1.0×10^6^ cell/ml). Four hours later, the presumptive fertilized eggs (zygotes) were transferred into fresh M16 medium lacking sperm, and allowed to incubate for 1 day. At that point, the percentage that had developed to the 2-cell stage was determined.

### Statistics

Error figures in tables and error bars in graphs are standard errors (SEM, standard deviations of the mean). Tests of significance were carried out by 1-way ANOVA using Graphpad Software's Instat, V. 3.02 followed by the Turkey-Kramer Multiple Comparison Test.

## Results

**Figure 1:**
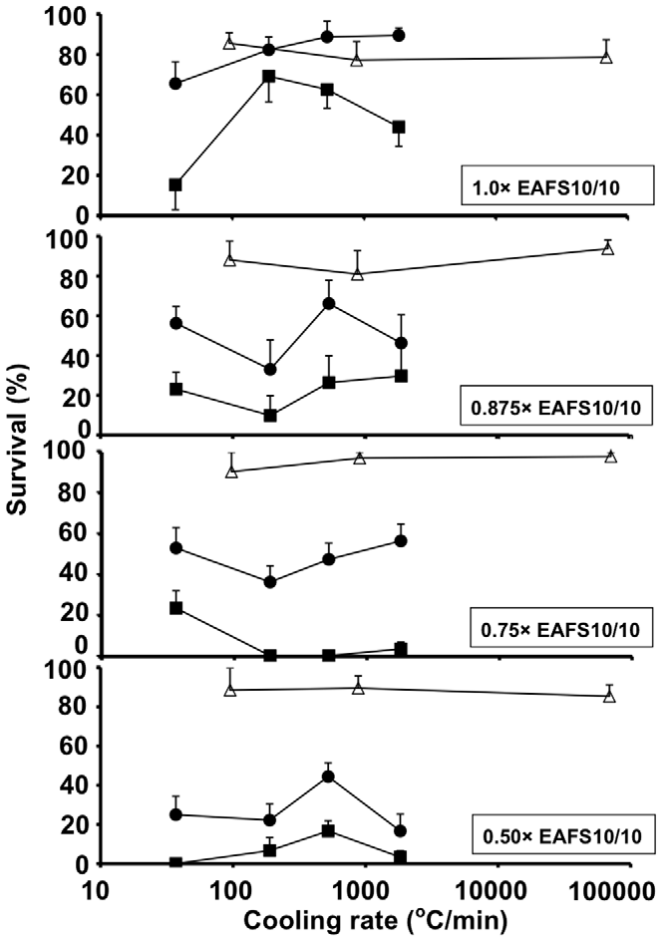
Survival of mouse oocytes as a function of the dilution of EAFS and the cooling and warming rate to and from −196°C. The dilutions were 1.0×, 0.875×, 0.75×, or 0.5×. The cooling rates ranged from 37°C/min to 69,250°C/min; the warming rates (°C/min) were 117,500 (Δ). 2,950 (•). and 2,170 (▪). The protocols are given in Tables 3 and 4. Survivals are based on morphological appearance and osmotic behavior after warming and after 1–2 hr incubation in M16 medium.

Figure 1 plots survival as a function of cooling rate and warming rate for oocytes suspended in 1× EAFS 10/10 (top panel) and in three dilutions of EAFS (0.875×, 0.75×, and 0.5×). For each dilution, the oocytes were cooled at 3 or 4 different rates ranging from a low of 37°C/min to a high of 69,250°C/min, and for each cooling rate they were warmed at three rates; namely, 2,170°C/min, 2,950°C/min, and 117,500°C/min. The survivals in this plot are based on the morphological normality, membrane intactness, and osmotic responsiveness of the oocytes after the post thawing treatment. Details are given in [19]. Table 5 gives details on the statistics.

**Table 5:** Survival based on morphological normality and osmotic responsiveness of mouse oocytes suspended in various dilution of EAFS10/10, and cooled and warmed at indicated rates in straws or on Cryotops.

Dilution of EAFS10/10	Device	Warming rate (°C/min)	Cooling rate (°C/min)						
			37	95	187	522	880	1827	69250
1.00×	Straw	2170	15.8±12.3 ^A, a^	-	69.4±12.7 ^B, a^	62.9±9.2 ^B, a^	-	44.4±9.5 ^AB, a^	-
			(4/28, N = 6)		(14/21, N = 6)	(22/35, N = 7)		(17/39, N = 8)	
	Straw	2950	65.8±10.7 ^A,^ * ^, a^	-	82.5±6.3 ^A,^ * ^, a^	88.9±7.7 ^A, a^	-	89.6±3.7 ^A,^ * ^, a^	-
			(24/38, N = 8)		(33/40, N = 8)	(27/31, N = 6)		(33/38, N = 6)	
	Cryotop	117500	-	82.5±6.9 ^A, a^	-	-	75.0±14.4 ^A, a^	-	91.7±6.3 ^A, a^
				(19/23, N = 4)			(18/24, N = 4)		(44/48, N = 8)
0.875×	Straw	2170	23.3±8.5 ^A, a^	-	10.0±10.0 ^A, b^	26.7±13.5 ^A, b^	-	30.0±16.2 ^A, ab^	-
			(7/30, N = 5)		(3/30, N = 5)	(8/30, N = 5)		(9/30, N = 5)	
	Straw	2950	56.7±8.5 ^A,^ * ^, ab^	-	33.3±14.9 ^A, b^	66.7±11.8 ^A, ab^	-	46.7±14.3 ^A, abc^	-
			(17/30, N = 5)		(10/30, N = 5)	(20/30, N = 5)		(14/30, N = 5)	
	Cryotop	117500	-	88.1±9.4 ^A, a^	-	-	81.0±11.7 ^A, a^	-	93.8±4.4 ^A, a^
				(32/35, N = 7)			(32/40, N = 7)		(45/48, N = 8)
0.75×	Straw	2170	23.3±8.5 ^A, a^	-	0.0±0.0 ^B, b^	0.0±0.0 ^B, b^	-	3.3±3.3 ^AB, b^	-
			(7/30, N = 5)		(0/23, N = 4)	(0/24, N = 4)		(1/30, N = 5)	
	Straw	2950	52.8±10.0 ^A, ab^	-	36.1±8.0 ^A,^†^, b^	47.2±8.0 ^A,^†^, b^	-	56.3±8.3 ^A,^ * ^, ab^	-
			(19/36, N = 6)		(13/36, N = 6)	(17/36, N = 6)		(27/48, N = 8)	
	Cryotop	117500	-	90.5±9.5 ^A, a^	-	-	97.1±2.9 ^A, a^	-	97.9±2.1 ^A, a^
				(36/40, N = 7)			(34/35, N = 7)		(47/48, N = 8)
0.50×	Straw	2170	0.0±0.0 ^A, a^	-	6.7±6.7 ^A, b^	16.7±5.3 ^A, b^	-	3.3±3.3 ^A, b^	-
			(0/30, N = 5)		(2/30, N = 5)	(5/30, N = 5)		(1/30, N = 5)	
	Straw	2950	25.0±9.4 ^A,^†^, b^	-	22.2±8.2 ^A, b^	44.4±7.0 ^A,^ * ^, b^	-	16.7±8.6 ^A, c^	-
			(9/36, N = 6)		(8/35, N = 6)	(16/36, N = 6)		(6/36, N = 6)	
	Cryotop	117500	-	88.6±11.4 ^A, a^	-	-	89.6±6.3 ^A, a^	-	85.4±5.8 ^A, a^
				(36/40, N = 7)			(35/38, N = 8)		(39/46, N = 8)
0.33×	Cryotop	117500	-	-	-	-	-	-	6.3±3.0 ^b^
									(3/48, N = 8)

The data are % survival ± SEM. The first sets of parentheses are the ratios of the number of surviving oocytes to the number frozen or vitrified. N is the number of replicate. Values with different superscripts were significantly different (P<0.05) by one-way ANOVA. Capital letters shows the differences of survivals with the same warming rate, same diluted vitrification solution and various cooling rate, and small letters shows those with same warming rate and same cooling rate and various diluted vitrification solutions.

* shows those with same cooling rate, same dilution of vitrification solution, and different warming rates of 2,170 or 2,950°C/min and ^†^shows that it was not possible to do stastical analysis because the other survival was 0%.

In Figure 1, the curves connecting the four closed circles depict the survivals of oocytes in straws cooled at 37, 187, 522, and 1,827°C/min and warmed at 2,950°C/min, the highest rate obtainable in straws. In full-strength EAFS, survivals are ≥65% with all four cooling rates as we reported previously [14], but they drop to ∼50%, 45%, and 25% as the EAFS is diluted to 0.875×, 0.75×, and 0.5×. If the warming rate is decreased just slightly to 2,170°C/min (closed squares), the drop in survival with dilution of the EAFS becomes exaggerated. In 0.5× EAFS, it approached 0%.

The curves connecting the three open triangles present a striking contrast. They depict the survivals of oocytes cooled on Cryotops at 95, 876, and 69,250°C/min and warmed at the highest rate attainable–−117,500°C/min. We see that with all four concentrations of EAFS, survivals range from 80 to 90% and are independent of the cooling rate.

**Figure 2:**
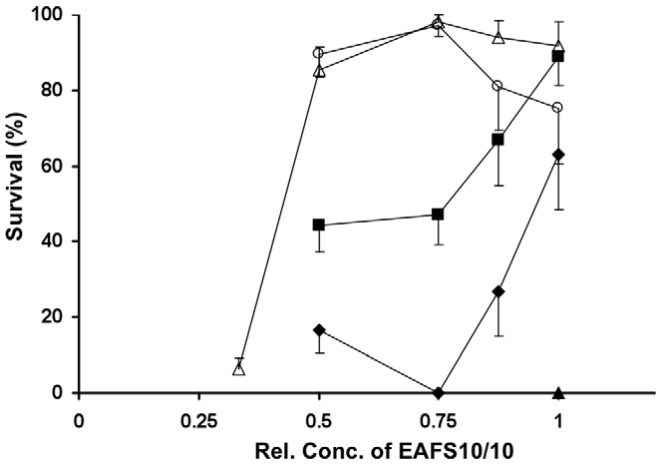
Survival of mouse oocytes vs. the relative concentration of the EAFS vitrification solution in which they were suspended. The relative concentrations ranged from 0.33× to 1×. The absolute concentrations of the individual solutes are given in Table 1 and 2. The suspensions were cooled to −196°C at rates ranging from 522 to 69,250°C/min and subsequently warmed at five different rates. The oocytes in the curves delineated by the open circles and triangles were warmed on Cryotops at the highest attainable rate of 117,500°C/min. The cells in the curves delineated by the closed symbols were warmed at lower rates between 610 and 2,950°C/min. CR and WR stand for cooling rate and warming rate. Symbol CR (°C/min) WR (°C/min) Δ 69,250 117,500 ○ 880 117,500 ▴ 69,250 610 ▪ 522 2,950 ♦ 522 2,170 Survivals are based on morphological appearance and osmotic behavior after 1–2 hr incubation in M16 medium.

Figure 2 shows morphological/osmotic survival as a function of the relative concentration of EAFS and includes the results for decreasing the concentration of EAFS from 1/2× to 1/3×. In that last case, we see that even with the highest warming rate of 117,500°C/min (open triangles), survival drops from ∼90% to 5%. Decreasing the concentration of solutes in the EAFS means decreasing the concentration of the non-permeating sucrose and Ficoll in the solutions and that in turn means that the oocytes are more hydrated in 1/2× EAFS than in full strength EAFS 10/10, and are more hydrated in 1/3× EAFS than in 1/2× EAFS. In the case of those in 1/3× EAFS, we presume that the high water content leads to the formation of enough intracellular ice during the subsequent cooling to kill them at that point. In the case of those cooled in concentrations of EAFS ≥0.5× the internal crystals that form during cooling can be prevented from recrystallizing to a lethal size by warming very rapidly.

Recrystallization is the conversion of a population of small ice crystals to a fewer number of larger crystals, a conversion that is driven by the former having a higher surface free energiy than the latter. This implies that if warming rates higher than the 117,500°C/min used here were attainable, it might be possible to achieve reasonably high survivals of mouse oocytes that are subjected to vitrification procedures in even more dilute solutions.

As indicated, the survivals in Figures 1 and 2 are based on morphological and osmotic normality. We have also obtained a measure of functional survival after vitrification in terms of the percentages of the oocytes that undergo fertilization and development to the 2-cell stage. Those percentages are shown in Table 6 where they are compared with morphological/osmotic survivals. In all these cases, the oocytes were warmed at the highest rate attainable (117,500°C/min). Several conclusions can be drawn with respect to morphological/osmotic survivals: [1] The average morphological survival (Column 3) is 89.3% [2]. Cooling rates ranging from 95°C/min to 69,000°C/min are essentially without effect [3], Relative EAFS concentrations ranging from 0.5× to 1× are almost without effect, and [4] these conclusions hold only when oocytes are warmed at the highest achievable rate.

**Table 6:** Morphological and functional survival of mouse oocytes suspended in various dilution of EAFS10/10, cooled at indicated rates on Cryotops, and warmed at 117,500°C/min.

Relative conc.	Cooling rate	% of Morphological Survival *		% of Oocytes	% of Morphol. Survivors
EAFS10/10	(°C/min)	+ 2 hr in M16	0 hr in M16	develop to 2-cell †	develop to 2-cell ‡
1×	69,000	91.7±6.3	91.7±4.5	80.8±3.8 ^a^	88.4
	880	75.0±14.4	89.4±5.5	54.2±9.2 ^ab^	62.9
	95	82.5±6.9	70.8±9.9	28.8±8.8 ^b^	39.3
0.875×	69,000	93.8±4.4			
	880	81.0±11.7	Not determined	Not detemined	Not determined
	95	88.1±9.4			
0.75×	69,000	97.9±2.1	96.9±3.1	76.0±4.3 ^a^	77.8
	880	97.1±2.9	96.7±3.3	63.3±9.5 ^ab^	66.7
	95	90.5±9.5	75.9±8.8	39.3±9.1 ^b^	52.5
0.5×	69,000	85.4±2.6	93.8±6.3	67.3±9.4 ^a^	71.4
	880	89.6±6.3	87.1±3.4	72.9±5.8 ^a^	97.1
	95	88.6±11.4	85.2±7.7	48.8±6.3 ^a^	59.4

^*^“Morphological survival" means an oocyte exhibiting normal osmotic responsiveness during the removal of EAFS and exhibiting normal morphology after 2 hr incubation in M16. That is, in the column labeled “+ 2 hr in M16", after the vitrified oocytes were warmed and the EAFS was removed, the eggs were incubated in M16 medium for 2 hr before being assessed for morphological normalcy. These are the survivals shown graphically in Figures 1 and 2. In the column labeled “0 hr in M16", as soon as the EAFS was removed, the eggs were scored for morphological survival. This was immediately followed by partial dissection of the zonae, and the mixing of eggs and sperm for IVF. In nearly all cases, each treatment was repeated 6 to 9 times with about 6 oocytes per repeat. In column of % of oocytes develop to 2-cell, values with different superscripts were significantly different (P<0.05) by one-way ANOVA. Letters show the differences of survivals with the same warming rate, same diluted vitrification solution and various cooling rates, and there is no significant differences with same warming rate and same cooling rate and various diluted vitrification solutions.

† The number of oocytes developing to 2-cell embryos after IVF/the number recovered after vitrification.

‡ Column 5/Column 4.

The picture changes somewhat when we compare functional survivals (columns 5 and 6). When the highest cooling rate and highest warming rate are combined, functional survivals range from 81% to 67% as the EAFS concentration is reduced from 1× to 0.5×. A cooling rate of 880°C/min is still relatively benign, but now a cooling rate of 95°C/min is decidedly damaging. The most likely explanation is that when oocytes are cooled at 95°C/min, the crystals that form intracellularly are large enough to be immediately lethal.

## Discussion

Subsequent to our initial finding published in 1972 [2], our laboratory has found that if mouse oocytes or preimplantation embryos suspended in a cryoprotectant like 1 M ethylene glycol are cooled at rates exceeding 2°C/min, they undergo lethal intracellular ice formation near −40°C, the homogeneous nucleation temperature of water [19]. As stated in the Introduction, one route to avoiding lethal IIF, and one being increasingly favored, is to suspend cells in much higher concentrations of cryoprotectants and cool them at much higher rates. Under such conditions, intracellular water can be converted into an innocuous vitreous or glassy state. Although the oocytes/embryos are cooled too rapidly in this procedure to undergo any osmotic dehydration during cooling, the nonpermeating solutes in vitrification solutions cause appreciable osmotic dehydration before cooling begins, and that dehydration in turn enhances the probability of vitrification.

The next step in the conceptual picture was our finding that if the water content of oocytes was reduced to between 40 and 23% of normal, only 12% and 0%, respectively, underwent visible IIF during cooling. For the others, the manifestations of internal ice appeared during warming [20]. We concluded that what we were seeing in these others during warming was the recrystallization or growth of small innocuous ice crystals that had formed during cooling to a lethal size during warming. We further found that the shorter the time spent during warming, the higher the temperature at which recrystallization of intracellular ice became evident [21]. And that led to the view that if the warming rate was high enough, intracellular recrystallization could be suppressed, and the oocytes or embryos would survive. We confirmed that hypothesis in two subsequent papers [16], [22]. The final step has been to extend this thinking to the possibility that with very high warming rates, one could substantially lower the concentrations of solutes in the vitrification medium. That has been the subject of the present paper.

We have identified three previous reports over the past 25 years of mouse embryos and two reports of oocytes surviving vitrification procedures in diluted vitrification media. The earliest of these by Rall [23] achieved good survival of 8-cell mouse embryos in a slightly diluted (0.85×) VS1 vitrification solution. Six years later, Leibo and Oda [24] obtained similar results for 8-cell mouse embryos suspended in a solution made with a low concentration of EG (2 M). and 7.5% polyvinylpyrrolidone When cooled at 1,250°C/min and warmed at 2,000°C/min, 76% developed into blastocysts. When the warming rate was slowed to 1,450°C/min, survival dropped to 26%. The other three [25]–[27] subjected mouse oocytes and embryos to very rapid cooling in or on devices like Cryotops and open and closed pulled straws, and by cooling in a liquid/solid nitrogen slush rather than LN_2_.. Cooling rates ranged from 32,000 to a reported 250,000°C/min and survivals ranged from 45% to 98% with the molarities of the vitrification media in the range of 2 to 5 M. For comparison, our survivals are 67–73% in 1.8 M medium [the sum of columns H–K for the 0.5× dilution in Table 1). All three groups attributed their success to the use of very high cooling rates, presumably because they believed that the ability of cells to vitrify in dilute solutions demands the use of the highest of cooling rates. We believe that the data in our present report oppose that view. Our data show that high percentages of oocytes in extensively diluted vitrification media will survive being cooled at moderate rates *provided* that the subsequent warming rate is exceedingly high.

Rall [23] was also the first to report that the survival of 8-cell embryos vitrified in straws in a full strength vitrification solution medium was highly dependent on the warming rate over a range of 10 to 200°C/min, but was totally independent of the cooling rate over a range of 20 to 2500°C/min. Strangely, that highly important finding was totally ignored the past 22 years until our reports in 2009 and 2011 [14], [22] and the current report. We believe there are two reasons. One, as mentioned, is that investigators prescribed to the reverse dogma. The second reason is that because of small thermal mass and small sample sizes, devices like Cryotops produce *both* very rapid cooling *and* very rapid warming. Consequently, when investigators used them, the contributions of the cooling and warming rates to survival were confounded since they took no specific steps to separate the effects.

Our findings are relevant both to fundamental cryobiology and to its applied aspects. On the fundamental side, they emphasize how critically survival in vitrification procedures depends on the use of the highest of warming rates to avoid or minimize the recrystallization of intracellular ice. They suggest that even higher warming rates might permit high survivals with even more dilute vitrification solutions.

On the applied and clinical side, the use of more dilute vitrification solutions may result in higher and more reproducible percentages of offspring developing from cryopreserved human oocytes or higher survivals of other cell types. For example, one adverse effect of high concentrations of protective solutes in oocytes appears to be hardening of the outer zona pellucida, a consequence of which is interference with the ability of sperm to enter the egg proper without the use of technically demanding ICSI (Intra-Cytoplasmic Sperm Injection). Another problem is that “open" devices like Cryotops, which were developed to achieve the very high cooling rates that were believed to be mandatory, place oocytes in direct contact with liquid nitrogen (LN_2_). There is concern that they could be contaminated by viable microorganisms in that refrigerant. Our finding that only moderate cooling rates are needed should eliminate that concern. By inserting the Cryotop tip with its adhering oocytes into its covering cap, it can be kept from contacting LN_2_ during cooling. The cap can then be removed while holding the device in cold vapor just above the LN_2_ to achieve extremely rapid subsequent warming when the now naked Cryotop is abruptly immersed into the sterile warming solution. Vanderzwalmen *et al.* [28] reported a similar approach to asepsis in 2009. Human blastocysts in vitrification solutions were placed in capillary VitriSafe devices which were hermetically sealed within straws and plunged into LN_2_. The double layered device slowed the cooling rate more than 10-fold from >20,000°C/min to <2000°C/min. To maintain a high rate of warming (>20,000°C/min), the straw constituting the outer surface was removed and the naked VitriSafe capillary plunged into the warming solution. They believed that the 10-fold lower cooling rate had to be compensated for by the use of a higher CPA concentration and/or a longer exposure to it. As we've noted, our findings differ; namely, one does not have to use a higher CPA concentration. In fact, our report deals with the successful use of concentrations of EAFS down to ½ of normal.

In stating that the findings in this report have clinical implications, we do not intend to imply that all the particulars we have found for mouse will apply to human oocytes. This may especially be the case for EAFS 10/10 since the acetamide it contains has been found to be somewhat toxic to the human egg. However, we believe that the matters of cooling rate vs. warming rate and warming rate vs. the dilution of vitrification solutions will apply. As we state, nearly all previously published papers have stressed the need to maximize the cooling rate, and many have attempted to increase the maximum. Our report shows that emphasis to be misplaced. It is the warming rate that needs to be maximized, and it is our hope that our paper will encourage some readers to attack the problem of obtaining even higher warming rates. If successful, we believe that will further facilitate the cryopreservation by vitrification procedures of difficult cell types like human oocytes and fish oocytes and embryos.

Beginning with our 2005 publication [19], we have based survival on the morphological/osmotic criteria summarized in Methods and Materials and detailed in [19]. This has left open the question of whether this reflects the functional survival of the oocytes. The current paper answers the question affirmatively to a considerable extent. The functional assay was the ability of the vitrified/warmed oocytes to undergo IVF and develop to the two cell stage. As we see in the far-right column of Table 6, between 63% and 97% of oocytes judged viable on morphological/osmotic grounds did develop to the 2-cell stage, provided that the cooling rate was 880°C/min or higher and the warming rate was 117,000°C/min.

To obtain these percentages of fertilization and development, we had to partially dissect the zona pellucida. In 1997, Nakagata *et al.* showed that partial zona dissection (PZD) of denuded B57BL/6j oocytes increased the success of IVF from 12% to 73–88% [29]. Two years later, An *et al.* [30] reported that the percentage of ICR 2-cell embryos derived by IVF after PZD that develop to blastocysts is the same or higher than that of 2-cell embryos with intact zonae. A more recent example is the 2011 report by Macas et al. [31] that the percentage of human oocytes that develop to blastocysts after IVF is the same for oocytes with intact zonae, those with partially dissected zonae, and those with zonae in which various-sized holes has been created by laser. Thus, the use of PZD is not a problem. What is a remaining problem is that vitrified ICR oocytes will not develop beyond the 2-cell stage. They behave as though they have a two-cell block, but we do not as yet know the explanation or how to resolve it.
